# Sex differences in donor-site morbidity after microvascular free tissue head and neck reconstruction

**DOI:** 10.1017/S0022215124001257

**Published:** 2025-01

**Authors:** Jordan I. Teitelbaum, David D. Choi, Kattia F. Moreno, Meredith E. Tabangin, Yash J. Patil

**Affiliations:** 1 Departments of Otolaryngology Head and Neck Surgery, University of Cincinnati Medical Center College of Medicine, Cincinnati, USA; 2 Division of Biostatistics and Epidemiology, Cincinnati Children's Hospital Medical Center, Cincinnati, Ohio, USA

**Keywords:** head and neck neoplasm, free tissue flaps, sex characteristics, quality of life

## Abstract

**Background:**

The purpose of this study is to investigate whether sex plays a role in donor-site dysfunction after head and neck reconstruction.

**Methods:**

In this retrospective case series, 76 patients were assessed for donor-site morbidity using the Short Form 36, Short Musculoskeletal Function Assessment, disabilities of the arm, shoulder, and hand, and lower-limb core scale. Differences by sex were compared using *t*-tests. Multivariable linear regression analysis was conducted to adjust for potential confounders.

**Results:**

Females observed significantly greater disability for the SF-36 mental component summary score with a mean of 45.9 (standard deviation 10.5) compared to males, with a mean of 51.8 (standard deviation 10.2), *p* = 0.02. Sex is significantly related to SF-36 mental component summary score after controlling for neuropsychiatric disease and tracheostomy status.

**Conclusion:**

Females reported significantly worse mental component scores compared to males undergoing free flap reconstruction of the head and neck.

## Introduction

Globally, head and neck cancer is a common malignancy accounting for more than 650,000 cases and 330,000 deaths annually.^1^ In the United States, head and neck cancer is responsible for 3 per cent of malignancies, with approximately 53,000 new cases annually. Head and neck cancer accounts for almost 15,000 deaths per year.^2^ Males are 2.7 times more likely to develop oral and pharyngeal cancer and 2.8 times more likely to die of this disease when compared to females. Males are 4.5 times more likely to develop larynx cancer and 4.8 times more likely to die of this disease when compared to females.^3^ Males are 1.26 times more likely to drink alcohol and 1.48 times more likely to smoke in the U.S., although these differences alone are insufficient to explain the threefold higher male head and neck cancer rates. Some hormone protective theories have been suggested to explain sex differences in head and neck cancer, but the evidence is not conclusive.^4^ Further studies are needed to elucidate these differences.

Treatment of head and neck cancer affects function and appearance. Surgical treatment of head and neck cancer can obviously cause deficits in speech, swallowing, cosmesis and breathing, which affect quality of life.^5^ The introduction of microvascular free tissue transfer in the 1970s greatly improved the treatment of head and neck cancer by providing better functional and aesthetic results after reconstruction.^6^ Free flap utilisation has become the standard for reconstruction after head and neck cancer resection, proving more versatile for wound closure, appearance, and restoration of function.^6,7^

With significant improvements in success rates for free flap reconstruction, attention has turned to donor-site morbidity.^8^ Many issues have been also associated with their use, including systematic recipient site and donor-site complications.^9^ Age, sex, alcohol use, tobacco use, pre-operative irradiation, comorbidity grade, cancer stage, operative time and reconstruction characteristics have all been studied to determine if they affect outcomes after microvascular reconstruction. Singh *et al*.^9^ noted that pre-operative morbidity and prior radiation are factors associated with the development and complications. Egestad *et al*.^10^ and Peters *et al*.^11^ concluded that age was a predictor of medical complications. Loupatazi *et al*.^12^ found that female sex and alcohol use were associated with severe complications.

Sex has been studied as a variable in the quality of life for head and neck cancer patients, but most results do not show significant differences.^10,11,13^ However, our group recently reported a trend in greater emotional disability among females.^14,15^ Although sex differences have been shown to have an effect in incidence, morbidity and mortality in head and neck cancer, few studies have examined the effect of sex as a factor of donor-site morbidity.

This study aimed to detect whether differences exist in donor-site morbidity between males and females following head and neck free tissue reconstruction.

## Methods

A retrospective study was designed to identify all head and neck reconstruction patients who specifically underwent free tissue reconstruction. Patients were excluded if they were unable or failed to complete all questions in the study tool. No sex, racial/ethnic, or educational level exclusion criteria were used. All patients were called by the senior author or members of the research staff to assess their willingness to participate in the survey. Surveys were then mailed to all patients in a single packet. If surveys were not returned, the patients were then called to request the completion and return of the surveys. Surveys were then completed either over the telephone or at their next follow-up visit.

We used general, site-specific, and disease-specific questionnaires to study post-operative patients. Four validated instruments were used: (general) Short Form 36 Health Survey (SF-36) with its two categories, physical component summary and mental component summary,^16,17^ (disease-specific) Short Musculoskeletal Function Assessment Questionnaire, which has two indices—the functional index and the bothersome index,^18,19^ and (site-specific) lower-limb core scale19--21and disabilities of the arm, shoulder and hand.^22^ Patients’ general well-being was assessed using the SF-36 questionnaire. This questionnaire evaluates eight distinct elements, including bodily pain, physical function, general health, vitality, mental health, social function, and role limitations secondary to physical and emotional problems, but it can be aggregated into two over-arching categories—mental health and physical health (physical component summary). In this questionnaire, lower scores demonstrate poorer quality of life.^16^

Short Musculoskeletal Function Assessment, disabilities of the arm, shoulder and hand and lower-limb core scale questionnaires examine functional outcomes. The Short Musculoskeletal Function Assessment evaluates general musculoskeletal function^18^ while the disabilities of the arm, shoulder and hand and lower-limb core scale specifically focus on the musculoskeletal function of the upper and lower extremities.^20,22^ The Short Musculoskeletal Function Assessment has two general indices—the functional index and the bothersome index, demonstrating the actual physical dysfunction and the extent to which patients are bothered by it respectively. For these questionnaires, higher scores indicate poorer function.^18^

The disabilities of the arm, shoulder and hand questionnaire is a generic measure of disability and symptoms related to any condition of any joint of the upper extremity. It is a 30-item questionnaire (21 physical function items, six symptom items and three social/role-function items) with two optional four-item modules to measure the effect of upper extremity disability on work (work module) or playing sports or musical instruments (sports and performing arts module). The questionnaire is designed so that higher scores indicate greater disability.^22^ The lower-limb core scale questionnaire consists of seven items addressing pain, stiffness, swelling and function, performed at an acceptable level to measure the effect of lower extremity disability.^20^ These site-specific questionnaires often differ in their length and time to complete, which can greatly affect their clinical utility. We think that the combination of these four questionnaires provides reliable results in the evaluation of donor-site morbidity.

The study protocol was approved by the Institutional Review Board at the University of Cincinnati College of Medicine (protocol 2013-5488). Between January 2009 and July 2014, 76 patients (21 years and older) were recruited from the University of Cincinnati Medical Center. One of two microvascular surgeons (author YJP and another surgeon) performed all reconstruction procedures.

### Statistical Analysis

All questionnaires and their respective sub-components were scored as previously described in the literature. Distributions of continuous variables were examined and summarised using means with standard deviation (SD) or medians with interquartile range. Percentages and frequencies were used to summarise categorical variables. Differences in the distributions of clinical characteristics (age, type of insurance, Charlson Comorbidity Index, length to completion (time in months from surgery to survey), flap location (donor site), flap type, squamous cell carcinoma (SCC), malignancy, cancer stage, adjuvant therapy, diagnosis of chronic pain not related to cancer, neuropsychiatric disease at the time of survey (anxiety, depression and/or bipolar) percutaneous endoscopic gastrostomy (PEG) at the date of surgery and the date of survey, tracheotomy at date of surgery and date of survey, and recurrence were examined by sex using chi-square or Fisher's exact tests for categorical data and *t*-test or Wilcoxon rank sum test for continuous data. Differences in mean survey scores by sex were compared using a *t*-test. We used multivariable linear regression to adjust for potential confounders of the relationship between sex and survey scores. Stepwise backward elimination was used for model parsimony. *P*-values less than 0.05 were considered statistically significant. Least squares mean with 95 per cent confidence intervals are reported. All statistical analyses were performed using SAS version 9.4 (SAS Institute, Cary, North Carolina, USA).

## Results

Eighty-four consecutive patients underwent head and neck reconstruction with microvascular free tissue transfer. Eight patients who were deceased or unable to be reached were excluded. The remaining 76 patients completed the study (88 per cent). Donor sites included radial forearm (*n* = 24, 31.6 per cent), latissimus (*n* = 21, 27.6 per cent), fibula (*n* = 19, 25.0 per cent) and scapula (*n* = 12, 15.8 per cent). All patients completed SF-36 and Short Musculoskeletal Function Assessment. Additionally, the site-specific questionnaire, disabilities of the arm, shoulder and hand, was used in the radial forearm, scapula, and latissimus free flaps post-operatively while patients with fibula free flaps completed the lower-limb core scale.

The mean patient age was 63 years old (SD 11.0, range 25–84; Table 1). Forty-eight (63.2 per cent) males and 28 (36.8 per cent) females were included. All patients in this study were cis gendered based on self-reporting. Sixteen patients (21 per cent) were being treated for a recurrence at the time of the study. The median time between surgery and completion of the survey was 13 months (interquartile range 6.5, 32.0). All patients required reconstruction for oropharyngeal defects. Seventy-two (94.5 per cent) patients had cancer diagnoses. Benign diagnoses included ameloblastoma (1), cocaine-induced oronasal fistula (1) and fractures (2). Three patients had sarcoma, while individual cases had myoepithelial, mucoepidermoid, adenoid cystic, oncocytic, adenocarcinoma and acinic cell carcinomas. All other patients had SCC. Sixty (82.2 per cent) patients underwent chemoradiation therapy. No patients experienced flap loss. No major complications related to the donor site required re-operation.

**Table 1.** Demographics and Clinical characteristics by sex

Characteristics, N = 76	Total	Female (*n* = 28)	Male (*n* = 48)	*P*-value
Age, Mean (SD)	63.0 (11.0)	64.25 (8.1)	62.25 (12.4)	0.40
Insurance				0.64
Public/None	27 (35.5)	9 (32.1)	18 (37.5)	
Private	49 (64.5)	19 (67.9)	30 (62.5)	
Charlson Comorbidity Index	5.2 (2.1)	4.79 (1.4)	5.48 (2.4)	0.12
Length Completion (m), Median [IQR]	13 [6.5, 32.0]	16.0 [9.0, 32.0]	11.5 [5.0, 31.0]	0.44
Flap Location (donor site)				0.81
Latissimus/Scapular	33 (43.4)	11 (39.3)	22 (45.8)	
Radial/OsteoRadial	24 (31.6)	10 (35.7)	14 (29.2)	
Fibula	19 (25.0)	7 (25.0)	12 (25.0)	
Flap type				0.70
Soft Tissue	44 (57.9)	17 (60.7)	27 (56.2)	
Soft Tissue and Bone	32 (42.1)	11 (39.3)	21 (43.8)	
				
Pathology: Malignancy	72 (94.7)	26 (92.9)	46 (95.8)	0.62**
TNM stage				0.10
< 3		8 (32.0)	7 (15.2)	
≥ 3		17 (68.0)	39 (84.8)	
Unknown		3	2	
Recurrence*	16/73 (21.9)	5/26 (19.2)	11/47 (23.4)	0.68
Adjuvant therapy*	60/73 (82.2)	21/26 (80.8)	39/47 (83.0)	0.81
Squamous cell carcinoma*	61/72 (84.7)	20/25 (80.0)	41/47 (87.2)	0.50
PEG on date of surgery	30 (39.5)	11 (39.3)	19 (39.6)	0.98
Tracheotomy on date of surgery	55 (72.4)	20 (71.4)	35 (72.9)	0.89
Neuropsychiatric disease	38 (50.0)	16 (57.1)	22 (45.8)	0.34
Chronic pain	4 (5.3)	2 (7.1)	2 (4.2)	0.62**
PEG on date of survey	14 (18.4)	4 (14.3)	10 (20.8)	0.48
Tracheotomy on date of survey	8 (10.5)	1 (3.6)	7 (14.6)	0.25**
*Missing: recurrence *n* miss = 3, Adjuvant therapy *n* miss = 3, Squamous cell carcinoma *n* miss = 4				
**Fisher's exact test				

Distributions of all other clinical variables including age, type of insurance, Charlson Comorbidity Index, donor site, length of completion, donor site, flap type, malignancy, cancer stage, adjuvant therapy, chronic pain, neuropsychiatric disease (none *vs* one or more), SCC, PEG on date of surgery, tracheotomy on date of surgery, PEG on date of survey, tracheotomy on date of survey, and recurrence were similar between females and males (Table 1). Questionnaire scores were normally distributed. Female patients observed lower scores for the SF-36 mental component summary score with a mean and standard deviation (SD) score of 45.9 (10.5) compared to males, with a mean and SD of 51.8 (10.2) (*p* = 0.02). SF-36 physical component summary, disease-specific (Short Musculoskeletal Function Assessment bothersome index and functional index), and site-specific (lower-limb core scale, and disabilities of the arm, shoulder and hand) scores were similar by sex (Table 2).

**Table 2.** Summary of study surveys score by sex

Outcomes by sex	Overall	Female	Male	*P*-value
General (100 = better health)	N = 76	N = 28	N = 48	
SF36 PCS Optum (Physical), Mean (SD)	41.1 (10.4)	42.2 (10.1)	40.4 (10.6)	0.49
SF36 MCS Optum (Mental), Mean (SD)	49.6 (10.6)	45.9 (10.5)	51.8 (10.2)	0.02
Disease-specific (100 = worse health)	N = 76	N = 28	N = 48	
SMFA Functional Index, Mean (SD)	19.6 (15.7)	21.3 (17.0)	18.7 (14.9)	0.48
SMFA Bothersome Index, Mean (SD)	23.4 (18.1)	24.7 (19.8)	22.7 (17.2)	0.64
				
	Overall	Female	Male	P-value
Location-specific	N = 57	N = 21	N = 36	
Arm: DASH, Mean (SD) (100 = worse health)	20.8 (17.2)	20.5 (18.5)	21.0 (16.6)	0.91
Median	15.8 [7.5, 30.8]			
Range	0–64.2			
	N = 19	N = 7	N = 12	
Lower Limb: LLO Standardised	87.1 (16.8)	82.7 (22.3)	89.7 (13.1)	0.4
Mean (SD)				
(100 = better health)				
Median	91 [82, 100]			
Range	36–100			
	N = 19	N = 7	N = 12	
Lower Limb: LLO Normative Score Mean (SD)	47.5 (12.3)	44.3 (16.4)	49.3 (9.5)	0.4
Median [IQR]	50 [44, 57]			
Range	10–57			

SF-36 (Short Form 36 Health Survey); SF-36 PCS (physical component summary) and SF-36 MCS (mental component summary),

SMFA (Short Musculoskeletal Function Assessment) Questionnaire; FI (functional index); BI (bothersome index).

LLCS (lower limb core scale)

DASH (disabilities of the arm, shoulder, and hand)

IQR (interquartile range)

In addition to sex differences in SF-36 mental component summary scores (female *vs* male beta estimate (standard error): −6.56 (2.3)), multivariable regression model results showed that patients who had a tracheotomy on the date of the survey and/or one or more neuropsychiatric disease had lower SF36 mental component summary scores (indicating greater disability). For least square means, 95 per cent confidence intervals for females were 46.2 (42.4, 50.0) *vs* males 51.6 (48.7, 54.5); having a tracheotomy 39.4 (32.4, 46.4) *vs* no tracheotomy on the date of survey 49.8 (47.5, 52.2); and one or more neuropsychiatric diagnoses 42.4 (38.2, 46.5) *vs* none 46.9 (42.3, 51.5). Sex is significantly related to SF-36 mental component summary after controlling for neuropsychiatric disease and tracheotomy status, such that males have higher SF-36 mental component summary scores.

**Figure 1.**
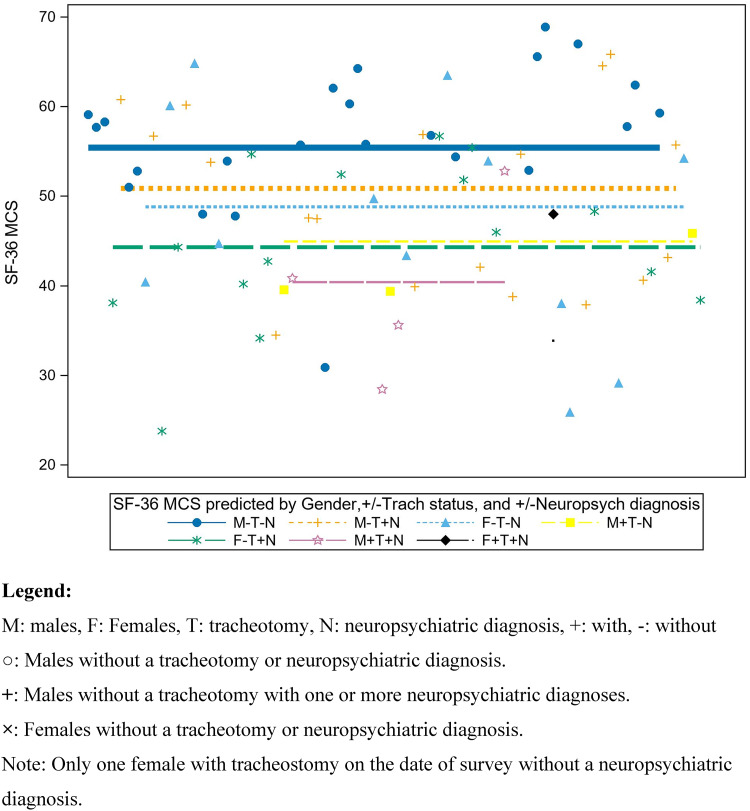
Regression Model: SF-36 MCS (mental component summary) and the regression lines predicted by sex, tracheotomy status and neuropsychiatric diagnoses.

Figure 1 illustrates the observed SF-36 mental component summary scores, and the regression lines predicted by sex, tracheotomy status and neuropsychiatric diagnoses. Males without a tracheotomy or neuropsychiatric diagnosis had higher SF-36 mental component summary scores, followed by males without a tracheotomy with one or more neuropsychiatric diagnoses and females without a tracheotomy or neuropsychiatric diagnosis.

## Discussion

The main objective of this study was to evaluate sex-based differences in donor-site morbidity after microvascular tissue transfer for head and neck reconstruction. We utilised four clinically validated questionnaires including general, disease-specific, and site-specific surveys: SF-36, Short Musculoskeletal Function Assessment, and disabilities of the arm, shoulder and hand/lower-limb core scale, respectively.^16,18,20,22^ Understanding factors that affect donor-site dysfunction or its perception will aid pre-operative counselling, flap selection and post-operative care.

Sex has been studied as a factor in prognosis, morbidity, and as a predictor of complications after surgery in oesophageal, urological, colon and other types of cancer. While females have a better prognosis than males after esophagectomy,^23^ they have a higher rate of complications after cystectomy.^24^ In head and neck cancer, Loupatatzi *et al*.^12^ found that being female seemed to affect the presence and/or severity of complications after microvascular free flap reconstruction. This finding suggests that sex differences exist after head and neck reconstructive surgery, however more studies are needed to examine these differences.

Sex and its effect on quality of life (QoL) have been examined in multiple studies. While some studies reported a slightly lower scores for women in many dimensions of QoL, the literature on sex difference has shown inconsistent results.^25^ Studies performed in developing countries have shown low female scores, which could be more related to the level of education and/or social or marital status rather than to gender itself.^13^ Some reports have found other factors that affect health-related quality of life more than sex, such as smoking,^10^ age,^11^ or disease-related variables like site, stage, treatment, and comorbidity. Site and stage have the biggest effect on QoL for head and neck cancer.^25^ Although health surveys in the general population show higher rates of symptoms, physical illness, and depression in women, studies in cancer concerning health related to QoL do not show a consistent difference between men and women.^25^ There is a paucity of data regarding how sex as a factor could influence head and neck reconstruction. The role of sex as a factor affecting morbidity after free tissue transfer reconstruction of head and neck patients remains unknown.

In this study, we evaluated the degree of deficit in upper or lower extremity donor sites following free tissue transfer reconstruction using four validated instruments and showed that sex is associated with the SF-36 mental component summary. In previous reports, the senior author has shown that there is a higher subjective dysfunction related to the donor site (upper and lower extremities) and these patients were significantly more bothered by this dysfunction than normal populations after free-flap reconstruction^14,15^

In this sample, we performed a comparison of scores of all domains between females and males and we only found that they significantly differ in SF-36 mental component summary. There are only a few studies that evaluated the sex differences in head and neck morbidity after Free Tissue Transfer.^12^ We did not find a sex difference in physical well-being, subjective dysfunction of the donor-site area (lower-limb core, disabilities of the arm, shoulder and hand) or in being bothered (bothersome index) by this dysfunction. In addition, we considered other variables that could have influenced these results as such age, type of insurance, Charlson Comorbidity Index, length to completion (time in months from surgery to survey), flap location (donor site), flap type, SCC, malignancy, cancer stage, adjuvant therapy, diagnosis of chronic pain not related to cancer, neuropsychiatric disease at the time of survey (anxiety, depression and/or bipolar), PEG at the date of surgery and date of survey, tracheotomy at date of surgery and date of survey, and recurrence. After controlling for these characteristics, sex is significantly related to the SF-36 mental component summary. This result agrees with previous reports which found that females tend to have more difficulty handling stress and pain and report decreased QoL during cancer treatment.^26,27^

- Free flap utilisation has become the standard for reconstruction after head and neck resection
- Attention has turned to donor-site morbidity
- There is a paucity of data regarding how sex could influence head and neck reconstruction
- We demonstrated significant greater disability for female SF-36 mental component after controlling for cofounders

The SF-36 mental component summary is a measure of mental health status which includes four domains: vitality, emotional role functioning, social functioning, and mental health. Studies suggest that the perception of pain in head and neck cancer patients is heightened in women.^28^ Pain is a domain that is evaluated in the SF-36 physical component summary which were similar in our population. We also evaluated chronic pain as a pre-existing diagnosis and did not find differences between females and males. We postulate that one or multiple domains in the SF-36 mental component summary are differentially affected by cancer surgery and treatment. Further studies are required to determine exactly which domains are affected.

We demonstrated in this study that females can be an independent predictor factor for worse mental health in patients after reconstruction with free flaps. We cannot conclude they are bothered by donor-site dysfunction or that discontent in female patients is related to their donor-site morbidity. Additional studies are needed.

To our knowledge, this is the first study designed to compare sex differences in donor-site morbidity after tissue transfer for head and neck cancer reconstruction. It is important to identify factors that are predictors of poor physical and psychological outcomes after head and neck cancer reconstructive surgery. For future studies, sex should be considered as an additional feature and should be evaluated with validated questionnaires that examine QoL in head and neck cancer patients.

## Limitations

This study included some limitations. Survey administration was completed at variable intervals after surgery. This limitation is inherent in survey studies. The skin graft size was not evaluated. All patients with fibula free flap or radial forearm free flap underwent skin grafting at the donor site, but the surface area was not recorded. The administration of multiple questionnaires made successful, timely completion challenging. These results may be limited by small sample sizes as well.

## Conclusion

Female patients reported worsened subjective mental assessment compared to male patients undergoing free flap reconstruction of the head and neck. Sex may be a factor to consider when counselling patients on options for head and neck reconstruction. To our knowledge, this is the first study designed to compare the sex differences in donor-site morbidity after tissue transfer for head and neck reconstruction.
